# Establishment of a novel cell cycle-related prognostic signature predicting prognosis in patients with endometrial cancer

**DOI:** 10.1186/s12935-020-01428-z

**Published:** 2020-07-20

**Authors:** Jinhui Liu, Jie Mei, Siyue Li, Zhipeng Wu, Yan Zhang

**Affiliations:** 1 Department of Gynecology, The First Affiliated Hospital of Nanjing Medical University, Nanjing, 210029 Jiangsu China; 2 Department of Oncology, Wuxi People’s Hospital Affiliated to Nanjing Medical University, Wuxi, 214023 Jiangsu China; 3 Department of Urology, The Affiliated Sir Run Run Hospital of Nanjing Medical University, Nanjing, 211166 China; 4 Department of Gynecology and Obstetrics, Wuxi Maternal and Child Health Hospital Affiliated to Nanjing Medical University, No. 48, Huaishu Road, Wuxi, 214000 Jiangsu China

**Keywords:** Endometrial cancer, Cell cycle, Prognostic model, TCGA, GSEA

## Abstract

**Background:**

Endometrial cancer (EnCa) ranks fourth in menace within women’s malignant tumors. Large numbers of studies have proven that functional genes can change the process of tumors by regulating the cell cycle, thereby achieving the goal of targeted therapy.

**Methods:**

The transcriptional data of EnCa samples obtained from the TCGA database was analyzed. A battery of bioinformatics strategies, which included GSEA, Cox and LASSO regression analysis, establishment of a prognostic signature and a nomogram for overall survival (OS) assessment. The GEPIA and CPTAC analysis were applied to validate the dysregulation of hub genes. For mutation analysis, the “maftools” package was used.

**Results:**

GSEA identified that cell cycle was the most associated pathway to EnCa. Five cell cycle-related genes including HMGB3, EZH2, NOTCH2, UCK2 and ODF2 were identified as prognosis-related genes to build a prognostic signature. Based on this model, the EnCa patients could be divided into low- and high-risk groups, and patients with high-risk score exhibited poorer OS. Time-dependent ROC and Cox regression analyses revealed that the 5-gene signature could predict EnCa prognosis exactly and independently. GEPIA and CPTAC validation exhibited that these genes were notably dysregulated between EnCa and normal tissues. Lower mutation rates of PTEN, TTN, ARID1A, and etc. were found in samples with high-risk score compared with that with low-risk score. GSEA analysis suggested that the samples of the low- and high-risk groups were concentrated on various pathways, which accounted for the different oncogenic mechanisms in patients in two groups.

**Conclusion:**

The current research construct a 5-gene signature to evaluate prognosis of EnCa patients, which may innovative clinical application of prognostic assessment.

## Background

Endometrial cancer (EnCa) ranks fourth in menace within women’s malignant tumors. In 2015, the American Cancer Society statistics found 10,170 deaths among 54,870 new cases with EnCa, which proved that the mortality rate of EnCa had increased significantly in the past 20 years. The average age of patients was 63 years old, about 90% of patients were over 50 years old, and only 20% of patients could be diagnosed before menopause [1]. Although increased numbers of studies have been conducted, EnCa still lacks early and noticeable symptoms, hence need to be effectively screened and managed [2–5].

Cell cycle is tightly associated with the growth and proliferation of cancer cells. Growing numbers of research have proven that genes can change the process of tumors by regulating the cell cycle, thereby achieving the goal of targeted therapy. For instance, Wu et al. uncovered that PRC1 could change the proliferation of oral squamous cell carcinoma by controlling the cell cycle [6]. Guo et al. found that KAI1 overexpression inhibited cell cycle in nasopharyngeal carcinoma cells [7]. Sun et al. found that CDC45 promoted papillary thyroid cancer development through controlling cell cycle [8]. Aspirin has been found to participate in the cell cycle arrest of oral squamous cell carcinoma, which may be used in therapeutic approaches [9].

In EnCa, cell cycle is a hot research direction. Shyam et al. found that centchroman induced cell-cycle arrest in human EnCa cells [10]. Qiu et al. discovered that JQ1 inhibited tumor growth by mediating PTEN/PI3K/AKT axis in EnCa [11]. Zhou et al. found that carfilzomib induced G2/M cell cycle arrest in EnCa cells [12]. Therefore, this study will extensively screen out cell cycle genes that were related to EnCa, providing different kinds of directions to treat EnCa.

## Material and method

### Acquisition of data

Transcriptional data and the corresponding EnCa clinical information were achieved from the TCGA database [13]. Platform Illumina HiSeq RNA‐seq [14] proceeded them and contained 552 EnCa patient samples and 35 normal tissues. Then 520 samples were get after integrating clinical information. These specimens were divided into the training cohort (n = 260) and the testing cohort (n = 260) randomly. The training cohort was applied for the prognostic signature establishment, and the testing and entire cohorts were used for validation of the prognostic signature.

### Gene set enrichment analysis (GSEA)

GSEA was conducted in the molecular signatures database (MSigDB) (http://software.broadinstitute.org/gsea/index.jsp), which provided hallmark gene sets to predict biological processes between normal and EnCa samples [15]. Next, we analyzed the expression levels of 32704 mRNAs in EnCa samples and normal tissues. The software running parameters are set to: replacement type selection phenotype, data set into gene name selection no, expression database selection gene cluster file, phenotypic tag selection: Tumor vs Normal,replacement parameters 1000 times, False discovery rate (FDR) < 0.01 were set as the cutoff.

### Identification of prognosis-related genes and their features

Univariate Cox regression, LASSO regression and multivariate Cox regression analyses were employed to explore the prognostic values of cell cycle-related genes in predicting EnCa patients’ overall survival (OS). In the univariate Cox regression analysis, genes were identified to be potential prognostic genes when *P value* was < 0.05. LASSO-penalized and multivariate Cox analysis were further conducted for further screening and narrowing prognostic genes. Hazard ratios (HRs) and regression coefficient were calculated for each hub gene, and five satisfactory genes were ultimately extracted. The gene alteration types and frequency of hub genes were exhibited by the cBioPortal tool [16].

### Construction of the gene‐related prognostic model

The prognostic signature for OS assessment of EnCa patients was the combination of each optimal prognostic transcriptional expression level multiplying relative regression coefficient weight calculated from the multivariate model according to the following formula:

$${\text{Risk Score}}\left( {\text{patient}} \right) = \mathop \sum \limits_{i} {\text{Coefficient}}\left( {{\text{mRNA}}_{i} } \right) \times {\text{Expression}}\left( {{\text{mRNA}}_{i} } \right)$$

All patients in the training cohort were classified into low- and high-risk groups based on the median of risk scores. The Kaplan–Meier survival curves of two groups and the ROC curve for OS evaluation were plotted to determine the sensitivity and specificity of the 5-gene signature [17]. Cox multivariate analysis including several clinical characteristics of EnCa patients was conducted as well to check the independency of the prognostic signature without clinical characteristics.

### Validation of the prognostic signature

By comparing the patient’s risk score in the testing and entire cohort with the cut-off value obtained from the training cohort, each patient was categorized as the low- or high-risk group. Kaplan–Meier curve, time-dependent ROC and multivariate cox analysis were also conducted. Furthermore, the subgroup survival analysis was performed according to different clinicopathological characteristics.

### Construction of nomogram based on the 5-gene signature

Nomogram and calibrate curves were established by the “rms” package in R language. The correctness was determined to check the consistency index between actual observation frequency and predicted probability. Next, we showed the predicted and observed results in the calibration curve to visualize the performance of the nomogram, and the 45° line represents the best prediction.

### Validation of the hub genes

GEPIA website was used to validate the cell cycle-related genes expression levels between EnCa and normal samples [18]. Besides, to further validate the protein levels of these five hub genes, the CPTAC analysis in UALCAN was applied [19].

### Mutation analysis

To compare the mutational loading between two groups, mutation annotation format (MAF) based on the TCGA cohort was functioned by the “maftools” package [20].

### Clinical specimens

We total collected 11 EnCa tissues and paired normal tissues in this research and all the patients were recruited by the Wuxi Maternal and Child Health Hospital Affiliated to Nanjing Medical University. The clinicopathological details were shown as Additional file 1: Table S1. Ethical approval for the study was granted by the Clinical Research Ethics Committee, Wuxi Maternal and Child Health Hospital Affiliated to Nanjing Medical University, and our research was conducted in accordance with the Declaration of Helsinki.

### Total RNA extraction and quantitative real-time PCR analysis

We used TRizol reagent (Thermo Fisher Scientific, Waltham, MA, USA) to extract total RNA from tissue samples and Agilent Bioanalyzer 2100 (Agilent Technologies, Santa Clara, CA, USA) with RNA 6000 Nano kit to evaluate the integrity of extracted RNA. We used a high-capacity cDNA reverse transcription kit (Thermo Fisher Scientific) to react with the extracted RNA to synthesize single-stranded complementary DNA from RNA, and then used the SYBR Green PCR kit (Thermo Fisher Scientific) for real-time quantification. Record the cycle threshold (Ct) of each gene. The relative expression of the target gene was calculated using the 2 − ΔΔCt method. All program steps of real-time quantitative RT-PCR (qRT-PCR) are performed in accordance with the instructions provided by the manufacturer. Primer sequences for five hub genes and GAPDH were shown in Table 1.

**Table 1:** Primer sequences for five hub genes and GAPDH

Gene	Primer sequences
EZH2	Forward: CCCTGACCTCTGTCTTACTTGTGGA
	Reverse: ACGTCAGATGGTGCCAGCAATA
HMGB3	Forward: CCCAGAGGTCCCTGTCAATTT
	Reverse: CGATCATAGCGCACTTTATCTGC
NOTCH2	Forward: CAACCGCAATGGAGGCTATG
	Reverse: GCGAAGGCACAATCATCAATGTT
UCK2	Forward: CTGAGCCAGGATAGCTTCTACC
	Reverse: CATACACGGGGATCTGGACTG
ODF2	Forward: TGGAGGCGGAAATGGATGG
	Reverse: CCTTGTCAGGGTGTTGATGTC
GAPDH	Forward: ACCACAGTCCATGCCATCAC
	Reverse: TCTAGACGGCAGGTCAGGTC

## Result

### Functional pathway screening using GSEA

**Fig. 1:**
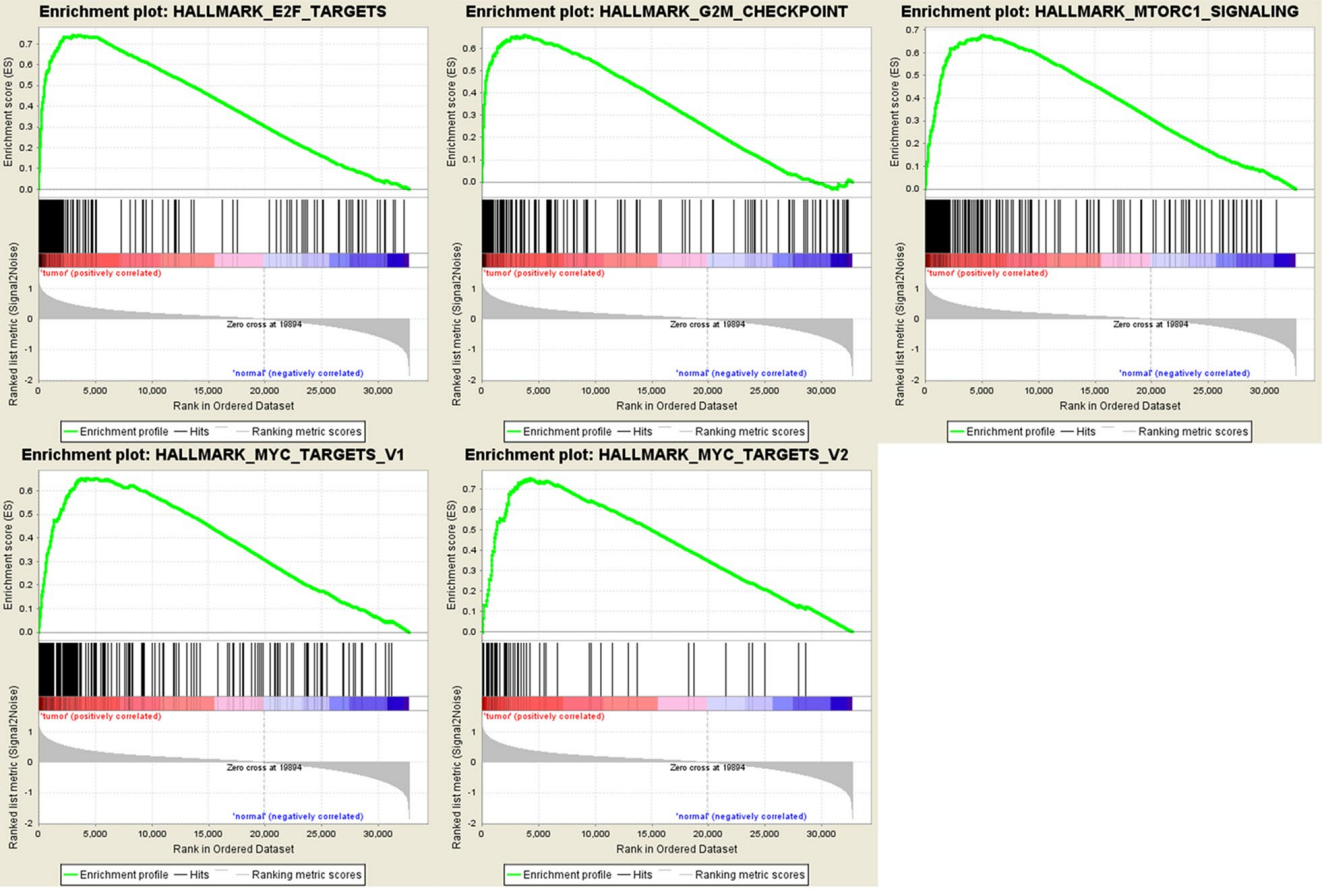
GSEA functioned to find the related functional pathway that might affect EnCa progression. Five significant pathways including E2F targets, G2M checkpoint, mtorc1 signaling, myc targets v1 and myc targets v2 were screened out

Clinical data from 587 samples, which contained 552 EnCa samples and 35 normal samples were obtained from the TCGA dataset. We performed GSEA to find the related functional pathway that might affect EnCa progression, which screened out 5 significant pathways that including E2F targets, G2M checkpoint, mtorc1 signaling, myc targets v1 and myc targets v2 (Fig. 1). G2M checkpoint was part of the cell cycle pathway, which was shown to be the most relevant.

### Establishment of cell cycle related genes prognostic model

We integrated transcriptional and clinical data to extracted 520 EnCa specimens. We analyzed 520 EnCa samples and obtained a total 193 related genes on the cell cycle pathway to investigate the association between cell cycle and the prognosis of EnCa patients. We randomly extracted 260 samples from a total of 520 samples, which we named as training cohort. We constructed a prognostic model in the training cohort and the univariate Cox regression analysis identified 13 genes according to the cutoff with *P *< 0.05. The 13 cell cycle genes were found to be associated with EnCa prognosis, which was further analyzed by LASSO Cox regression algorithm (Additional file 2: Figure S1A, B). Then multivariate Cox regression analysis was conducted for the risk signature. We established the prognostic signature and the risk scores were calculated for each sample. HMGB3, EZH2, NOTCH2, UCK2 and ODF2 were identified as significantly prognostic-related genes. The risk score was calculated as the followed formula: risk score = 0.000142772 * HMGB3 + 0.000762595 * EZH2 + 0.000100702 * NOTCH2 + 0.000430074 * UCK2 − 0.000400515 * ODF2.

**Fig. 2:**
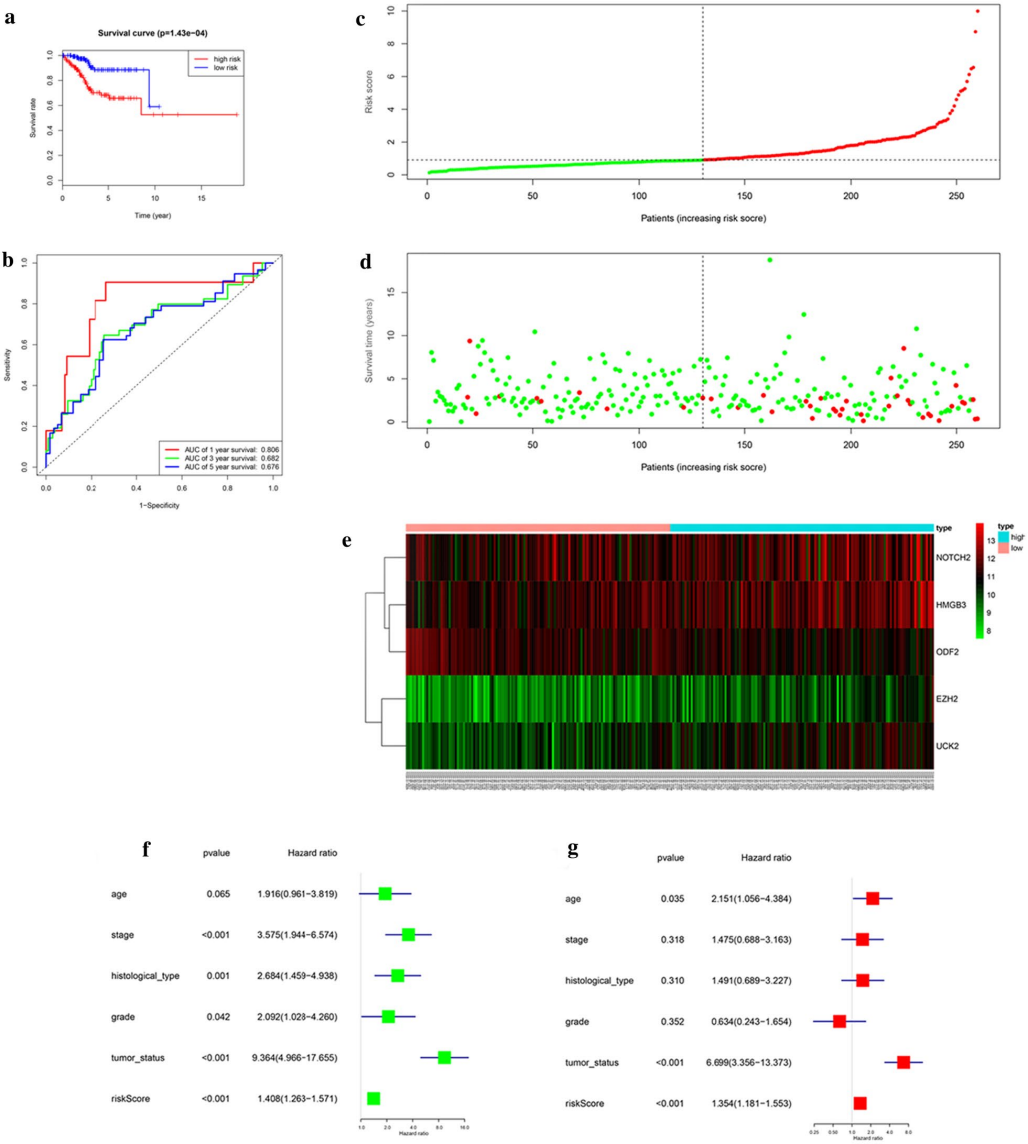
Prognostic model of the training cohort and risk signature with the 5 cell cycle-related hub genes. **a** Kaplan–Meier survival analysis of the low- and high- risk group patients in the training cohort. **b** ROC curve analysis according to the 1, 3, 5-year survival of the area under the AUC value. **c**, **d** The risk scores for all patients in the training cohort are plotted in ascending order and marked as low risk (blue) or high risk (red), as divided by the threshold (vertical black line). **e** The distribution of risk score, survival status, and the expression of 5 genes of each patient in training cohort by z-score, with red indicating higher expression and light blue indicating lower expression. **f** Univariate regression model. **g** Multivariate regression model

We used the median level of the risk score to classify the EnCa patients into low- and high-risk groups. Kaplan–Meier survival analysis of this model suggested that low-risk patients had notably preferable OS than high-risk patients (Fig. 2a). ROC analysis was demonstrated in Fig. 2b, AUC values for 1-, 3-, 5-year survival were 0.806, 0.682, 0.676, respectively. The risk score and survival status of this model were shown in Fig. 2c–e. Univariate and multivariate models were built, including risk scores and clinical factorsto confirm whether they were independent predictor of EnCa. The results showed that those prognostic models were indeed independent predictors for cell cycle pathway (Fig. 2f, g).

### Validation of the 5-gene signature

**Fig. 3:**
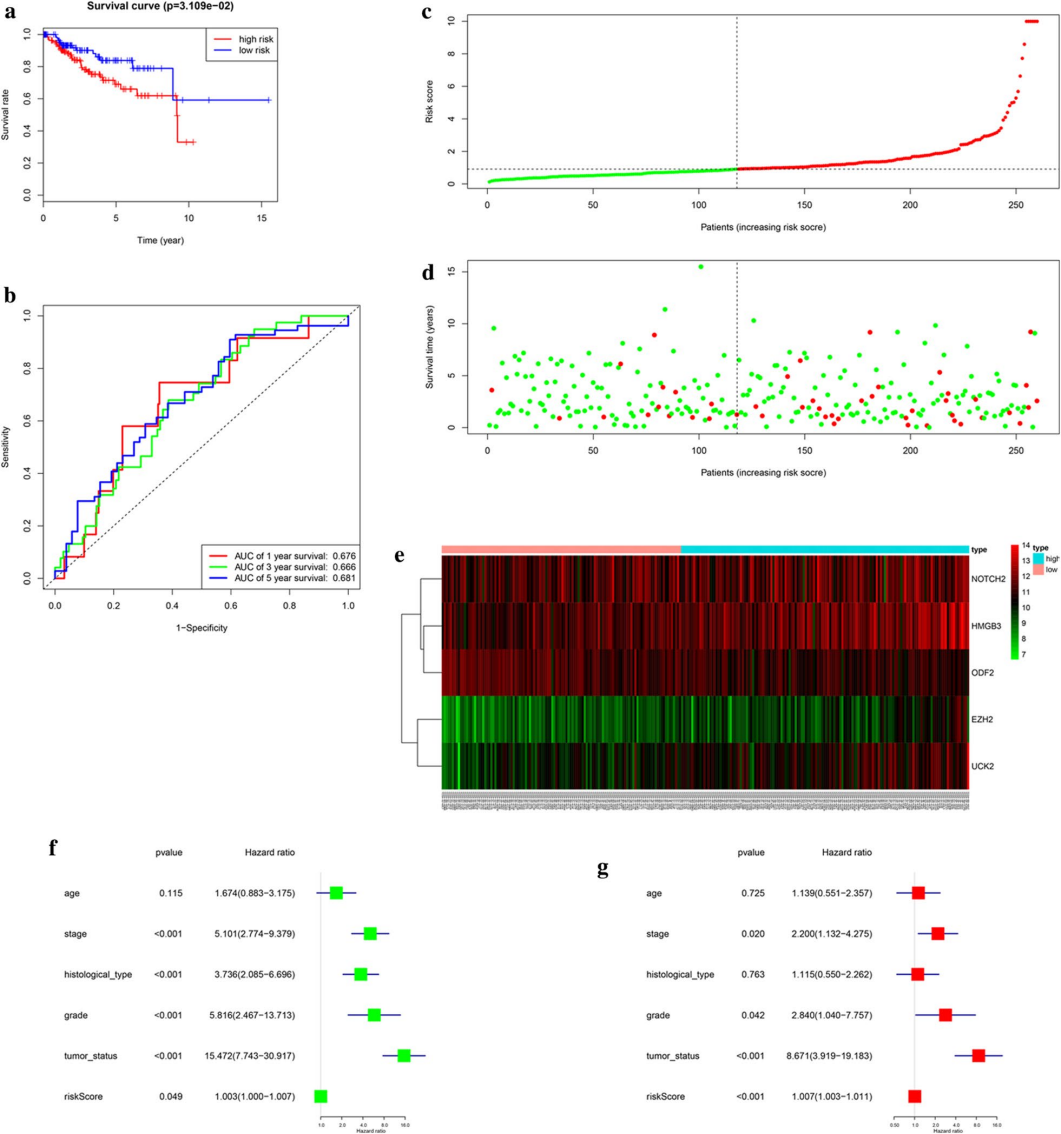
Prognostic model of the testing cohort and risk signature with the 5 cell cycle-related hub genes. **a** Kaplan–Meier survival analysis of the low- and high- risk group patients in the testing cohort. **b** ROC curve analysis according to the 1, 3, 5-year survival of the area under the AUC value. **c, d** The risk scores for all patients in the testing cohort are plotted in ascending order and marked as low risk (blue) or high risk (red), as divided by the threshold (vertical black line). **e** The distribution of risk score, survival status, and the expression of 5 genes of each patient in testing cohort by z-score, with red indicating higher expression and light blue indicating lower expression. **f** Univariate regression model. **g** Multivariate regression model

In order to testify the veracity of the 5-gene prognostic signature, we grouped the left 260 samples apart from the training cohort as the testing cohort, which was used to build another prognostic model. According to the median of risk score, samples were divided into low- and high-risk groups according to training cohort’ cut-off. Survival analysis exhibited that low-risk patients had remarkably better OS than high-risk patients (Fig. 3a). ROC curve analysis exhibited AUC values for 1-, 3-, 5-year survival were 0.676, 0.666, 0.681, respectively (Fig. 3b). Figure 3c–e also showed the risk score and survival status. The results of Univariate and multivariate Cox regression analyses combined the risk scores with clinical features exhibited moderate and independent prognostic power for cell cycle pathway (Fig. 3f, g). All of those conclusions were consistent with the results of training cohort, validating the reliability of our hypothesis that cell cycle was involved in the progression of EnCa.

**Fig. 4:**
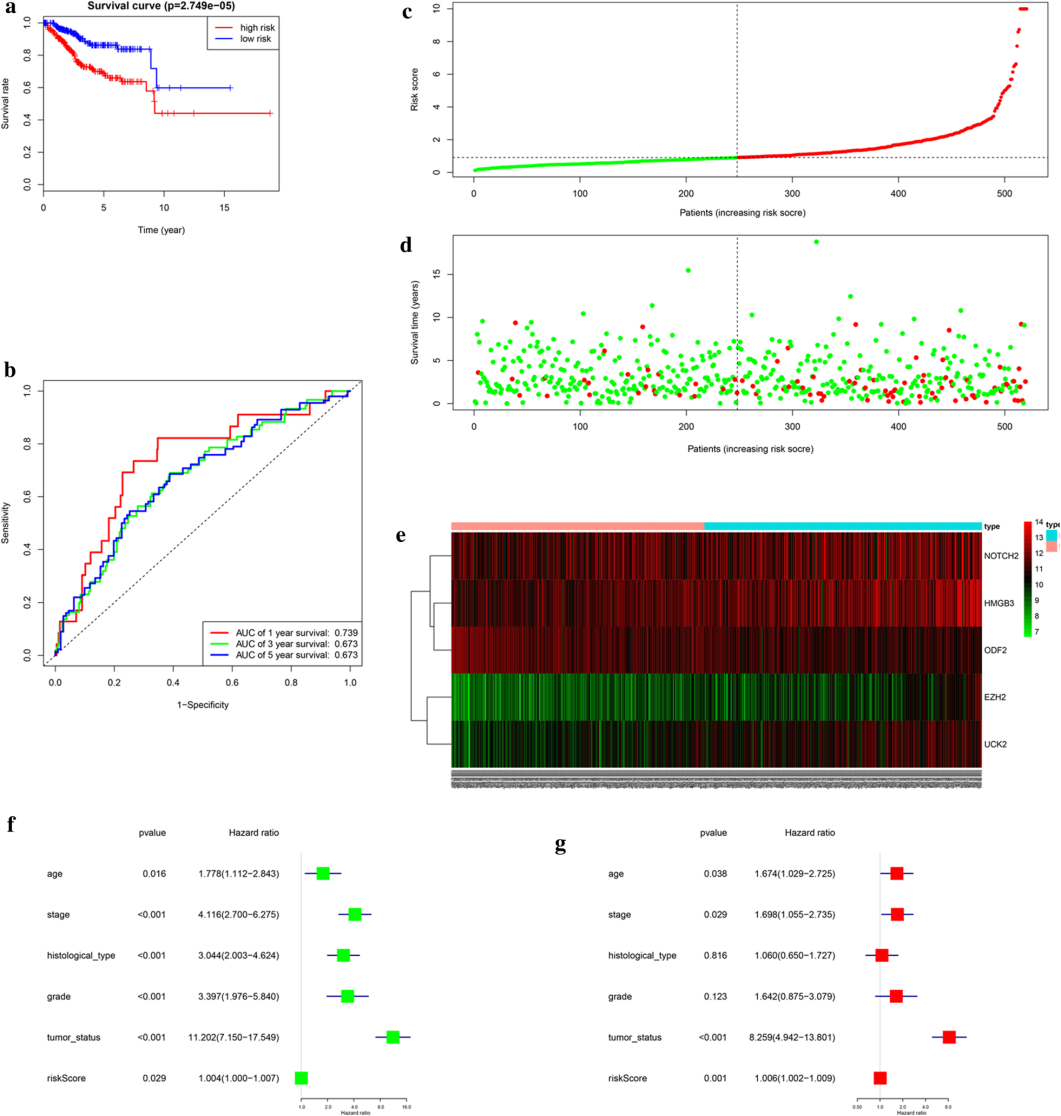
Prognostic model of the entire cohort and risk signature with the 5 cell cycle- related hub genes. **a** Kaplan–Meier survival analysis of the low- and high- risk group patients in the entire cohort. **b** ROC curve analysis according to the 1, 3, 5-year survival of the area under the AUC value. **c**, **d** The risk scores for all patients in the entire cohort are plotted in ascending order and marked as low risk (blue) or high risk (red), as divided by the threshold (vertical black line). **e** The distribution of risk score, survival status, and the expression of 5 genes of each patient in the entire cohort by z-score, with red indicating higher expression and light blue indicating lower expression. **f** Univariate regression model. **g** Multivariate regression model

We named the whole 520 samples as the entire cohort, which was used to build the complete prognostic model. Samples were also divided into low- and high-risk groups based on the median level of risk score and training cohort’ cut-off. Survival analysis indicated that low-risk patients had notably preferable OS than high-risk patients (Fig. 4a). ROC curve analysis exhibited AUC values for 1-, 3-, 5-year survival were 0.739, 0.673, 0.673, respectively (Fig. 4b). Figure 4c–e displayed the risk score and survival status which belong to the prognostic model. Univariate and multivariate Cox regression analyses which combined the risk scores and clinical features were exhibited that this prognostic signature was an independent predictor of EnCa as well (Fig. 4f–g). These results further validated the reliability of the established 5-gene prognostic signature.

### Hierarchical analysis of hub genes and clinical features

**Fig. 5:**
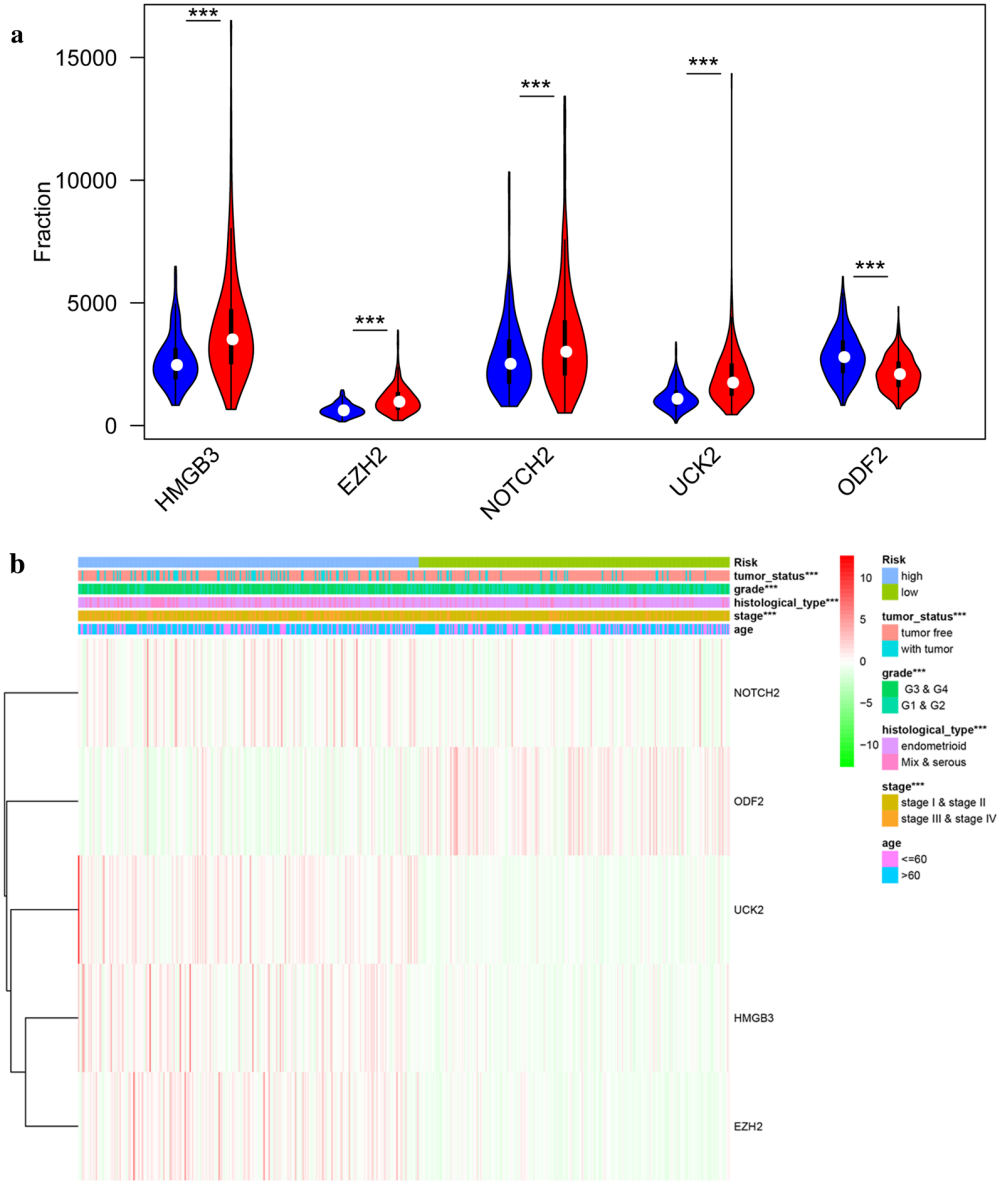
Validation of the 5 cell cycle-related hub genes **a** Expression levels of 5 significant cell cycle-related hub genes in high- and low-risk groups. **b** Heatmap showed the expression of the 5 cell cycle-related hub genes in high- and low-risk patients in the TCGA dataset associated with tumor status, grade, histological type, age, risk and stage. ***P < 0.001

Univariate and multivariate Cox proportional hazards regression analysis identified 5 cell cycle genes to be prognosis-related including HMGB3, EZH2, NOTCH2, UCK2 and ODF2. Significant differences were found in the expression levels of the five genes among the low- and high-risk groups (Fig. 5a). Besides, the expression of the 5 genes in low- and high-risk patients in the TCGA dataset was also demonstrated in the heatmap (Fig. 5b). We found significant differences between the high- and low-risk groups associated with tumor status, grade, histological type and stage. We deeply analyzed the relationship between the 5 genes and different clinical factors. We found they were significantly relevant. Furthermore, we analyzed the 5 genes for different clinical features, respectively. We found that expression levels of EZH2, HMGB3, NOTCH2 and ODF2 were significantly different in different grade groups (Additional file 3: Figure S2A–D). For different histological types, the expression levels of NOTCH2 and ODF2 were significantly different (Additional file 4: Figure S3A, B). For different age, the expression level of ODF2 was significantly different (Additional file 4: Figure S3C). For different tumor status, EZH2 and ODF2 expressed differently (Figure S4A-B). For different stages, EZH2, NOTCH2 and ODF2 expressed differently (Additional file 5: Figure S4C–E).

Then, the subgroup analysis was conducted based on histological type, grade, age, tumor status and stage. Next patients were stratified into endometrioid subgroups, grade G1&G2 subgroup, grade G3&G4 subgroup, stage III & stage IV subgroup, tumor-free subgroup, age > 60 subgroup and age ≤ 60 subgroup. For the patients in endometrioid subgroup, the survival time of patients in the high-risk group was remarkably shorter than that of patients in the low-risk group (Additional file 6: Figure S5A), consistent with the trends for the grade G1&G2 subgroup, grade G3&G4 subgroup, stage III & stage IV subgroup, tumor-free subgroup, age > 60 subgroup and age ≤ 60 subgroup. (Additional file 4: Figure S5B–G).

### Building predictive nomogram

**Fig. 6:**
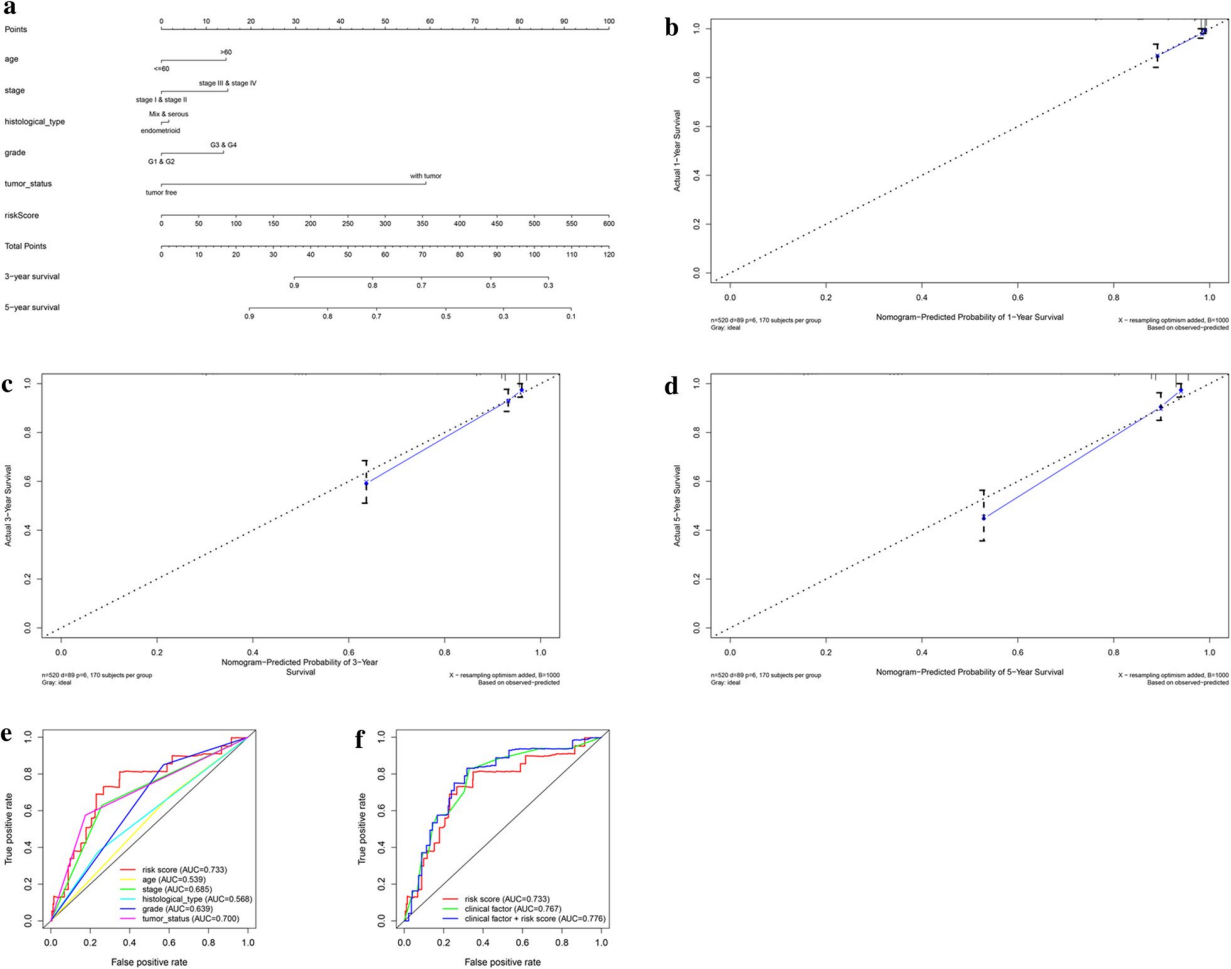
The nomogram to predict 1-, 3‐ or 5‐year OS and prognostic value of 5 genes in the entire set. **a** The nomogram for predicting the proportion of patients with 1-, 3‐ or 5‐year OS. **b–d** The calibration plots for predicting patient 1-, 3‐ or 5‐ year OS. Nomogram‐predicted probability of survival is plotted on the x‐axis; actual survival is plotted on the y‐axis. **e**, **f** Time-dependent ROC curve analyses of the 5-mRNA signature, age, tumor status, histological type and grade in the TCGA cohort

To achieve the goal of establishing a clinical strategy to predict the survival probability with EnCa patients, a nomogram was plotted using the TCGA cohort to evaluate the probability of the 1-, 3‐ and 5‐year OS. The predictors of the nomogram contained 6 prognostic factors including stage, age, histological type, grade, tumor status and risk score (Fig. 6a). The 45° line represented the best prediction. Calibration plots uncovered that the nomogram performed well (Fig. 6b–d). ROC curve analysis in Fig. 6e, f exhibited that the risk score AUC value of the model was 0.733, the clinical factors AUC value was 0.767, both remarkably higher than the clinical stage (AUC = 0.685), grade (AUC = 0.639), histological type (AUC = 0.568), tumor status (AUC = 0.700) and patients’ age (AUC = 0.539). Interestingly, when comprehensively conducted the ROC analysis based on the risk score with clinical features, the ROC curve was notably higher than each alone (AUC = 0.776). Principal component analysis based on the training, testing, and entire cohort displayed a different distribution pattern of low and high risk according to 5 cell cycle gene expression, indicating their difference in cell cycle aspect (Additional file 7: Figure S6A–C).

### Genetic alterations and expression of 5 hub genes

cBioPortal software showed the genetic alterations of 5 cell cycle-related genes. Additional file 8: Figure S7A, B showed that the 5 genes were altered in 89 (16%) of the 547 patients/548 samples; NOTCH2 and UCK2 showed most diverse alteration types, including amplification, missense mutation, deep deletion, and etc.

**Fig. 7:**
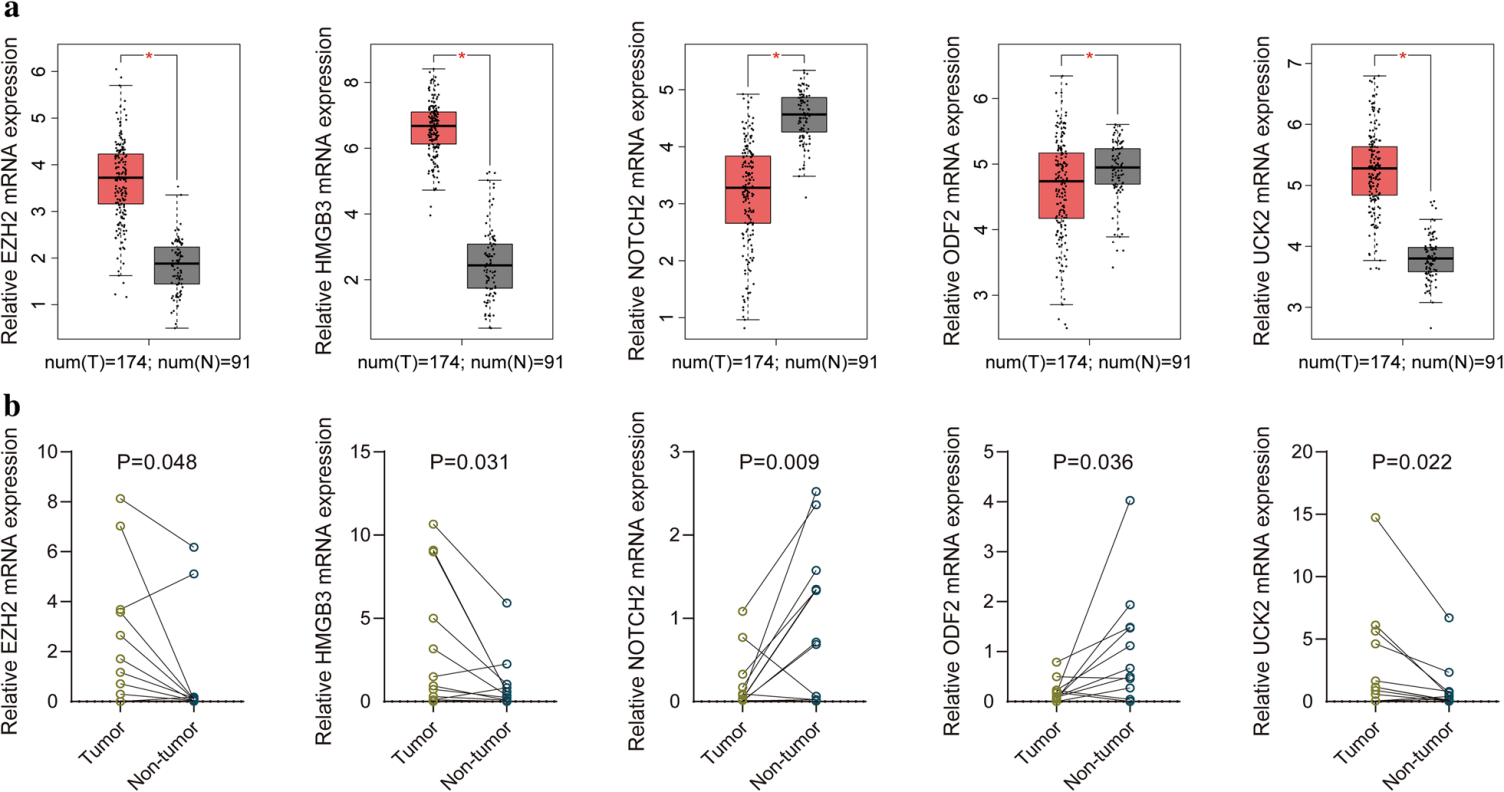
Validation of the 5 hub genes. **a** GEPIA validated the expression levels of the 5 hub genes between normal tissues and EnCa tissues. **b** Recruited cohort validated the expression levels of the 5 hub genes between normal tissues and EnCa tissues

**Fig. 8:**
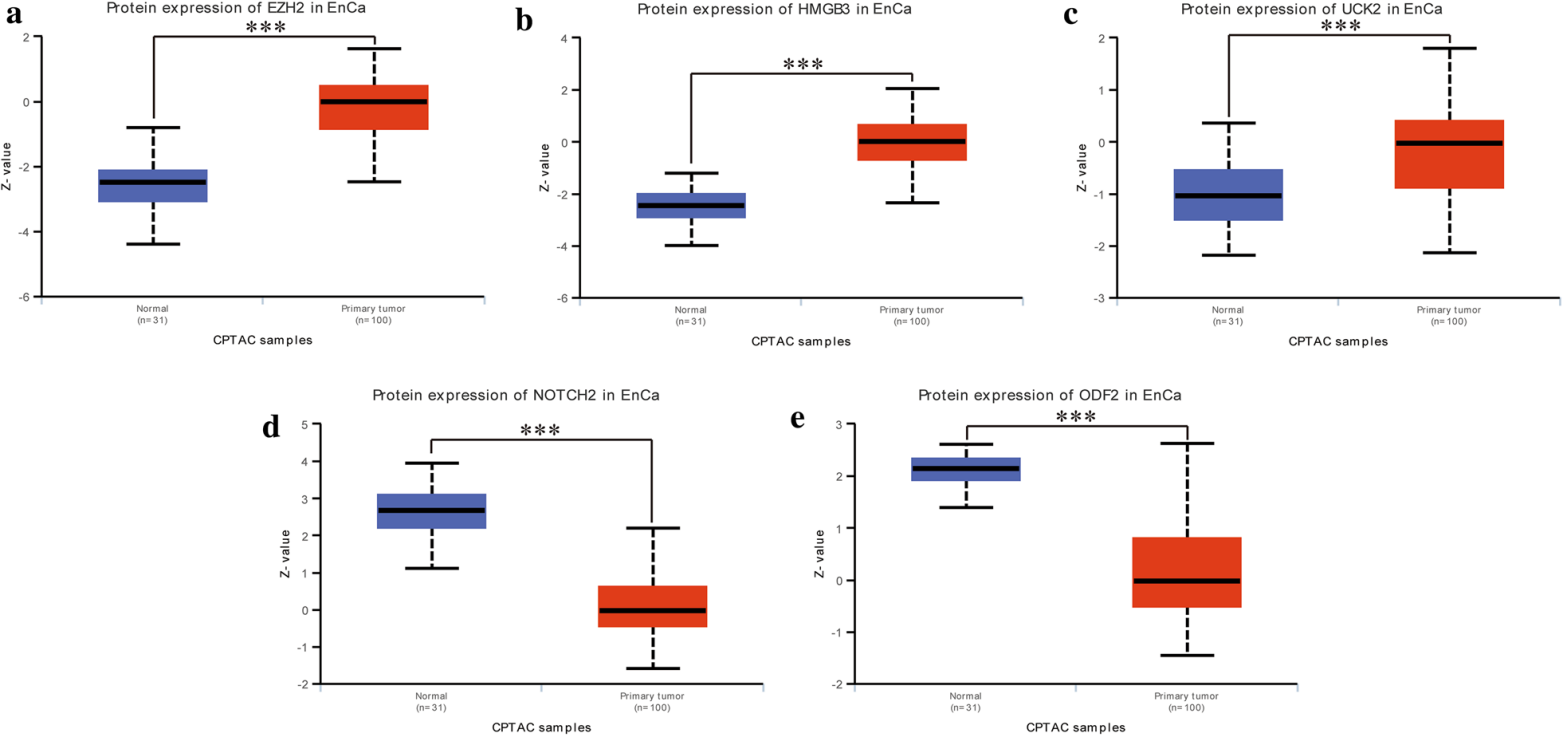
The protein expression difference of (**a**) EZH2, (**b**) HMGB3, (**c**) UCK2, (**d**) NOTCH2 and (E) ODF2. ***P < 0.001

GEPIA website validated the expression of the 5 cell cycle-related hub genes (Fig. 7a), Besides, our recruited cohort also validated the differential expression levels of the 5 hub genes between normal tissues and EnCa tissues (Fig. 7b). The AUC value of the 5 hub genes was shown in Additional file 9: Figure S8, reflecting the diagnostic efficacy. Together, these genes had an AUC value of 0.978, proving that they can efficiently distinguish normal tissues from cancerous tissues. EZH2, HMGB3 and UCK2 expressed higher in tumor compared with normal tissues, while NOTCH2 and ODF2 expressed lower in tumor compared with normal tissues. We also verified the protein levels of the above genes expression in the CPTAC database (Fig. 8a–e), and the protein levels of these genes were consistent with the results in GEPIA. Kaplan–Meier curves exhibited that overexpression of EZH2, HMGB3 and NOTCH2 were significantly associated with poor prognosis, while the lower expression of ODF2 was significantly correlated with poor prognosis (Additional file 10: Figure S9).

### Mutational loading between two groups based on the 5-gene signature

We next investigated whether EnCa with high-risk score was related to specific tumor mutation. Alteration landscape EnCa with high or low-risk scores were exhibited in Additional file 11: Figure S10. Ten genes were mutated in > 22% of samples with high-risk score: PTEN (50%), TP53 (50%), PIK3CA (45%), TTN (38%), ARID1A (37%), PIK3R1 (30%), KMT2D (26%), CTNNB1 (24%), CSMD3 (23%) and ZFHX3 (22%). While ten genes were mutated in > 25% of samples with low-risk score: PTEN (80%), ARID1A (54%), PIK3CA (53%), TTN (39%), PIK3R1 (32%), CTCF (32%), KMT2D (27%), MUC16 (27%), KRAS (25%) and ZFHX3 (25%). Specifically, lower rates of PTEN mutation, TTN mutation, ARID1A mutation, PIK3R1 mutation, ZFHX3 mutation and PIK3CA mutation in EnCa with high-risk score were found compared with EnCa with low-risk score.

### Identification of risk score associated biological pathways

**Fig. 9:**
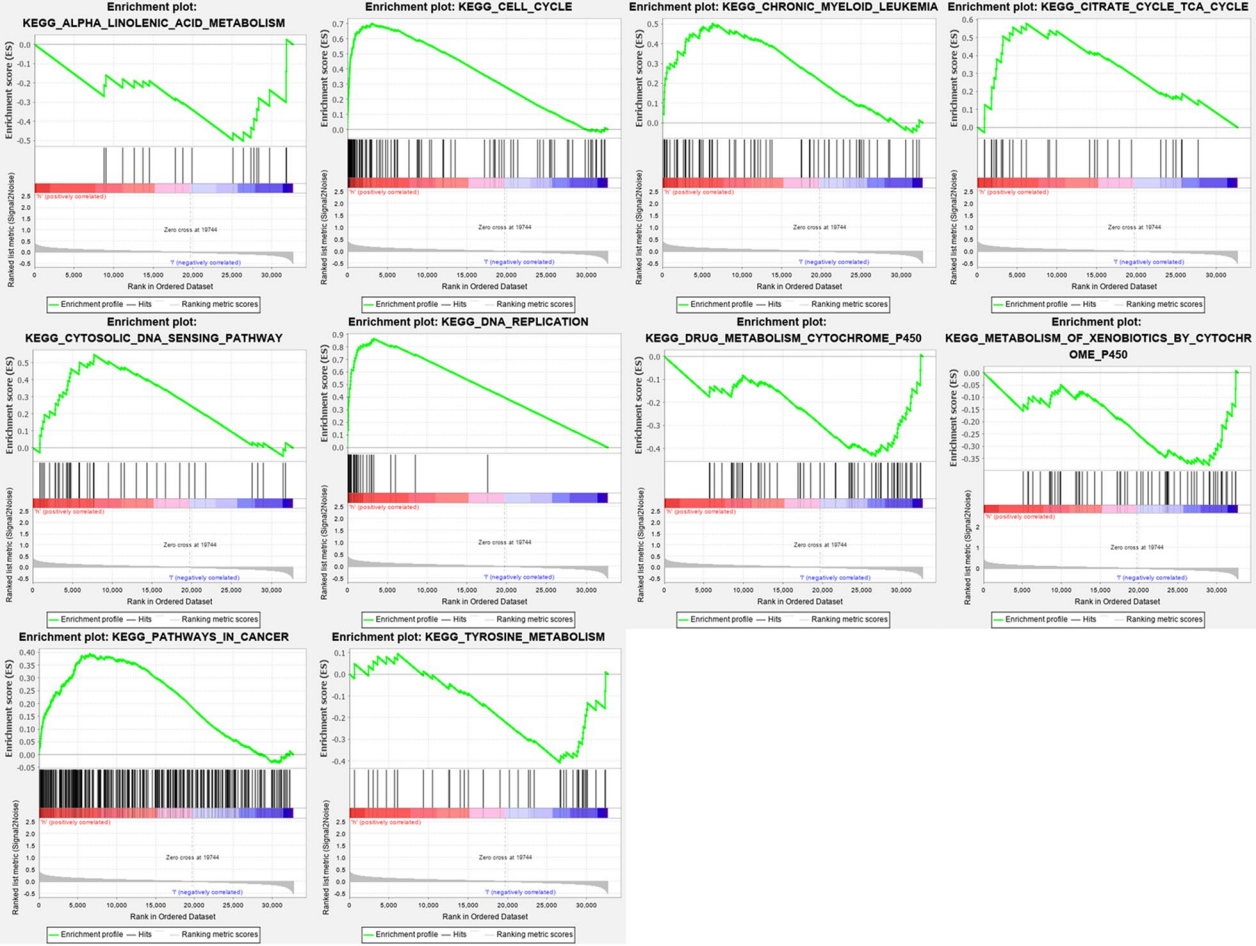
Gene set enrichment analysis in the TCGA database. Enrichment map were used for visualization of the GSEA results

GSEA further analyzed low and high-risk group samples, revealing the primary enrichment pathway. The samples of the high-risk group were mainly enriched in pathways such as cell cycle, DNA sensing pathway and myeloid leukemia, which was consistent with the results we obtained above. The samples of the low-risk group were mainly enriched in pathways such as tyrosine metabolism and alpha linolenic acid metabolism (Fig. 9).

## Discussion

EnCa is a widespread malignant tumor that threatens women’s lives worldwide, which usually occurs in postmenopausal women and is difficult to diagnose at the early stage [1]. Cell cycle is tightly associated with the growth and proliferation of cancer cells. Increasing numbers of research have proven that genes can change the process of tumors by regulating the cell cycle, thereby achieving the goal of targeted therapy. Therefore, we analyzed EnCa samples by GESA, cell cycle was found to be the most highly enriched pathway.

We next tried to develop a cell cycle-related prognostic signature. EnCa samples were then randomly divided into the training cohort and the testing cohort. We used the training cohort to establish a prognostic model by Cox and LASSO regression analysis, testing cohort and entire cohort were used for validation of prognostic signature. HMGB3, EZH2, NOTCH2, UCK2 and ODF2 were screened out. Although new biomarkers are discovered every day, the use of gene signature can highlight the most important in practical application. Compared to other established signatures to assess OS in EnCa [21, 22], our model was constructed and validated from more comprehensive cohorts and it seemed to be more convenient to be applied in clinical practice with fewer numbers of genes.

High mobility group box 3 (HMGB3) is a member of the high-mobility group box (HMGB) family. The HMG-Box subfamily acts significant roles in DNA replication, transcription, recombination and repair [23, 24]. HMGB3 has been widely researched in tumors. Research by Zhang et al. proved that overexpressed HMGB3 promoted proliferation and migration of cancer cells, accelerating the progression of colorectal cancer [25]. Studies by Gu et al. demonstrated that silencing HMGB3 expression suppressed breast cancer cell proliferation, thus inhibited tumor growth [26]. It has been proved that the regulation of HMGB3 by tumor suppressive miR-205-5p suppressed cancer cell aggressiveness and participated in prostate cancer progression [27]. HMGB3 was proved to promote the proliferation and invasion of glioblastoma cells as well [28]. However, there is no research on the role of HMGB3 in EnCa up to now, which should be further explored.

Enhancer zeste homolog 2 (EZH2) is the catalytic subunit of PRC2, which methylates Lys27 of histone H3, resulting in transcriptional repression of the target genes [29]. Wang et al. found that EZH2 played a tumor-suppressive role in K-Ras-mutation-driven lung adenocarcinoma [30]. Studies by Böhm et al. have shown that Loss of EZH2 at tumor invasion front was associated with an aggressive phenotype of cancer cells in colorectal cancer [31]. EZH2 has also been shown to promote hepatocellular carcinoma progression [32]. Interestingly, EZH2 has been shown to be an oncogene and promote EnCa progression by multiple studies, which is consistent with our findings [33–35]. For example, EZH2 mRNA and protein expression in EnCa specimens were significantly higher than in matched-normal tissue [36]. Besides, EZH2 siRNA in combination with taxanes produced more robust anti-tumor effects versus those induced by monotherapies [37].

NOTCH2 seems to be proven to be an oncogene by Xiu et al. [38] and the pathogenic effects were mainly mentioned in lung cancer [39]. Research by Devor et al. found that dysregulated miR-181c expression influenced the recurrence of endometrial endometrioid adenocarcinoma by regulating NOTCH2 expression [40]. Combined with our conclusions, this has become a direction worthy of further research.

Uridine-cytidine kinase 2 (UCK2) and ODF2 have not been studied in EnCa. UCK2 was proved to promote migration and invasion of hepatocellular carcinoma cells [41], which was also seen to be a latent diagnostic and prognostic indicator for lung cancer [42]. Overexpression of UCK2 was exhibited to be correlated with breast cancer progression and worse prognosis [43]. Yang et al. reported that ODF2 could maintain centrosome cohesion by restricting β-catenin accumulation [44]. ODF2 is also named as Cenexin 1, which is a molecular marker of mature centriole. ODF2 has been reported to be essential for maintaining proper centriole orientation and microtubule organizing [45, 46]. However, there is less research on ODF2 in tumors, it is a novel gene and it is worth our further exploration.

The somatic mutations in specific genes between low- and high-groups were examined by maftools package. The results exhibited that the various mutated genes could contribute to the different sore of genes in EnCa patients. It was uncovered that PTEN, ARID1A, PIK3R1 and ZFHX3 were important suppressors that participated in cancer development [47–50]. A study conducted by Chung et al. found that PIK3CA mutation was associated with cervical cancer in Hong Kong Chinese women [51].

In addition to the cell cycle pathway, GSEA also suggested that the samples of the high-risk group were mainly enriched in pathways such as DNA sensing pathway. The samples of the low-risk group were mainly enriched in pathways such as tyrosine metabolism and alpha linolenic acid metabolism. Research by Deng et al. proved that STING-dependent cytosolic DNA sensing promoted radiation-induced type I interferon-dependent antitumor immunity in immunogenic tumors [52]. Cheng et al. found tyrosine metabolism to be associated with esophageal squamous cell carcinoma [53]. Chamberland et al. found that alpha linolenic acid could downregulate the malignant potential of human and mouse colon cancer cells [54]. These research results prove that our GSEA analysis results are credible.

The current study also has several limitations. Although the number of samples in our research is currently the largest for EnCa, which is fewer than other cancer data sets. Besides, screened genes are not actually validated in clinical samples and cells. The specific mechanism will be designed in detail in future research.

## Conclusion

The current research found that the cell cycle pathway was associated with EnCa and screened for hub genes on the cell cycle pathway, which may be used as novel targets for the treatment of EnCa. Besides, a 5-gene prognostic signature was constructed based on cell-cycle related genes, which could be used for prognostic assessment for EnCa patients.

## Supplementary information

**Additional file 1: Table S1.** The clinicopathological parameters of EnCa patients involved in this research.

**Additional file 2: Figure S1.** Part prognostic model of the training cohort. (A-B) The coefficients calculated by LASSO.

**Additional file 3: Figure S2.** Expression levels of EZH2, HMGB3, NOTCH2 and ODF2 in different grade group. (A) EZH2, (B) HMGB3, (C) NOTCH2, (D) ODF2.

**Additional file 4: Figure S3.** (A-B) Expression levels of NOTCH2 and ODF2 in different histological type, (C) expression level of ODF2 in different age group.

**Additional file 5: Figure S5.** Survival time of patients in high-risk and low-risk group of different subgroups. (A) endometrioid subgroup, (B) grade G1&G2 subgroup, (C) grade G3&G4 subgroup, (D) stage III & stage IV subgroup, (E) tumor free subgroup, (F) age>60 subgroup, (G) age≤60 subgroup.

**Additional file 6: Figure S5.** Survival time of patients in high-risk and low-risk group of different subgroups. (A) endometrioid subgroup, (B) grade G1&G2 subgroup, (C) grade G3&G4 subgroup, (D) stage III & stage IV subgroup, (E) tumor free subgroup, (F) age>60 subgroup, (G) age≤60 subgroup.

**Additional file 7: Figure S6.** Principal component analysis of the training cohort, the testing cohort, and the entire EnCa cohort. (A) The training cohort, (B) The testing cohort, (C) The entire cohort.

**Additional file 8: Figure S7.** The gene mutation overview of 5 prognostic cell cycle-related genes in the TCGA EnCa patients. (A) Five genes were altered in 89 (16%) of the 547 patients/548 samples. (B) The summary of mutation types of 5 genes in EnCa patients.

**Additional file 9: Figure S8.** AUC value was used to identify the diagnostic efficacy of distinguishing normal and cancerous tissues.

**Additional file 10: Figure S9.** Kaplan-Meier curves of the EZH2, HMGB3, NOTCH2 and ODF2. The yellow line indicates samples with highly expressed genes (above best-separation value), and the green line designates the samples with lowly expressed genes (below best-separation value)

**Additional file 11: Figure S10** Alteration landscape for EnCa samples with high risk score and low-risk in the TCGA cohort. Lower rates of PTEN mutation, TTN mutation, ARID1A mutation, PIK3R1 mutation, ZFHX3 mutation and PIK3CA mutation in EnCa with high-risk score were found compared with EnCa with low risk score.

## Data Availability

All data are included in the article.

## Abbreviations

EnCa: Endometrial cancer
GSEA: Gene set enrichment analysis
MsigDB: Molecular signatures database
FDR: False discovery rate
OS: Overall survival
HRs: Hazard ratios
MAF: Mutation annotation format
Ct: Cycle threshold
qRT-PCR: Real-time quantitative RT-PCR
HMGB: High-mobility group box
EZH2: Enhancer zeste homolog 2
UCK2: Uridine-cytidine kinase 2
