# Supplementary material for: Establishment of a novel cell cycle-related prognostic signature predicting prognosis in patients with endometrial cancer
Source: Cancer Cell Int. 2020 Jul 20;20:329. doi: 10.1186/s12935-020-01428-z (PMC7372883; doi:10.1186/s12935-020-01428-z)
Supplement: Supplementary file 1 — Additional file 1: Table S1. The clinicopathological parameters of EnCa patients involved in this research. [file 12935_2020_1428_MOESM1_ESM.docx]

**Table S1. The clinicopathological parameters of EnCa patients involved in this research.**

| **Cohort ID** | **Age** | **FIGO stage** | **Lymphatic metastasis** | **Distant metastasis** | **Differentiated degree** |
| --- | --- | --- | --- | --- | --- |
| 20191113 | 50 | Ⅱ | No | No | Ⅰ-Ⅱ |
| 20191107 | 50 | IA | No | No | Ⅱ |
| 20190919 | 60 | Ⅱ | No | No | Ⅰ-Ⅱ |
| 20190628 | 64 | IA | No | No | Ⅱ |
| 20190617 | 67 | IIIC | Yes | No | Ⅱ |
| 20190603 | 53 | IA | N.A. | No | Ⅰ |
| 20190513 | 63 | IA | No | No | Ⅰ-Ⅱ |
| 20190506 | 64 | IA | No | No | Ⅲ |
| 20190429 | 60 | IA | No | No | Ⅰ-Ⅱ |
| 20190416 | 49 | IA | N.A. | No | Ⅰ-Ⅱ |
| 20190402 | 56 | IA | No | No | Ⅲ |
