# Supplementary figures and images for: Establishment of a novel cell cycle-related prognostic signature predicting prognosis in patients with endometrial cancer
Source: Cancer Cell Int. 2020 Jul 20;20:329. doi: 10.1186/s12935-020-01428-z (PMC7372883; doi:10.1186/s12935-020-01428-z)

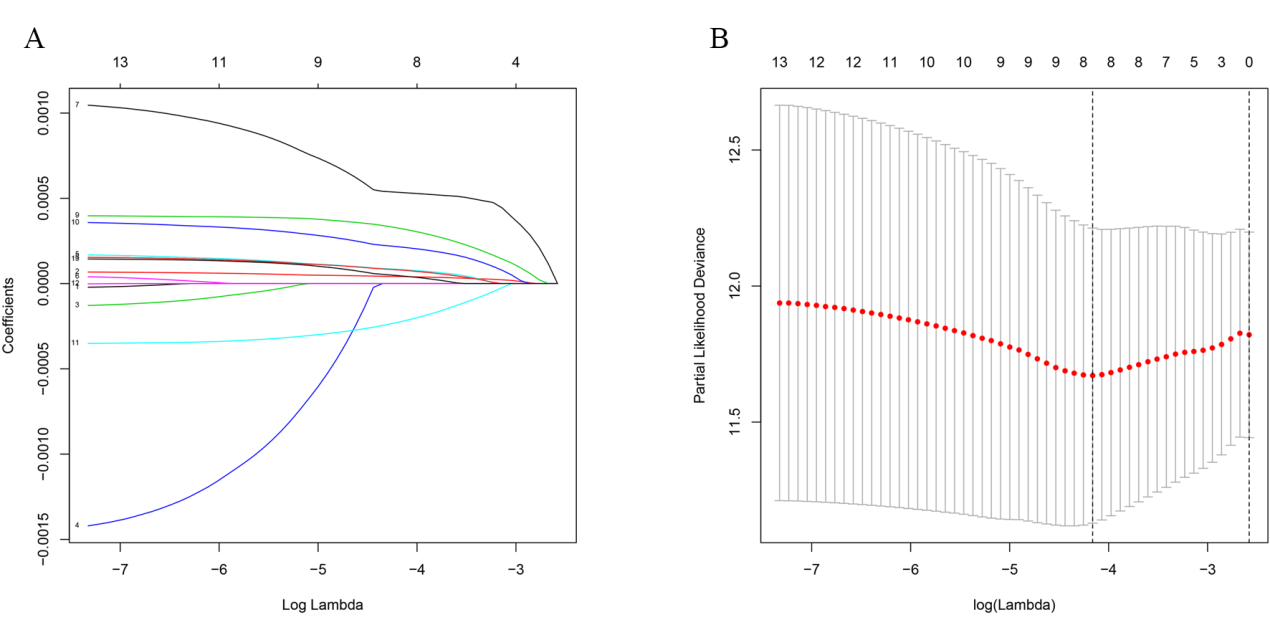
**Figure S1** Part prognostic model of the training cohort. (A-B) The coefficients calculated by LASSO.

Supplement: Supplementary file 2 — Additional file 2: Figure S1. Part prognostic model of the training cohort. (A-B) The coefficients calculated by LASSO. [file 12935_2020_1428_MOESM2_ESM.docx]
