# Supplementary material for: Establishment of a novel cell cycle-related prognostic signature predicting prognosis in patients with endometrial cancer
Source: Cancer Cell Int. 2020 Jul 20;20:329. doi: 10.1186/s12935-020-01428-z (PMC7372883; doi:10.1186/s12935-020-01428-z)
Supplement: Supplementary file 3 — Additional file 3: Figure S2. Expression levels of EZH2, HMGB3, NOTCH2 and ODF2 in different grade group. (A) EZH2, (B) HMGB3, (C) NOTCH2, (D) ODF2. [file 12935_2020_1428_MOESM3_ESM.docx]

**
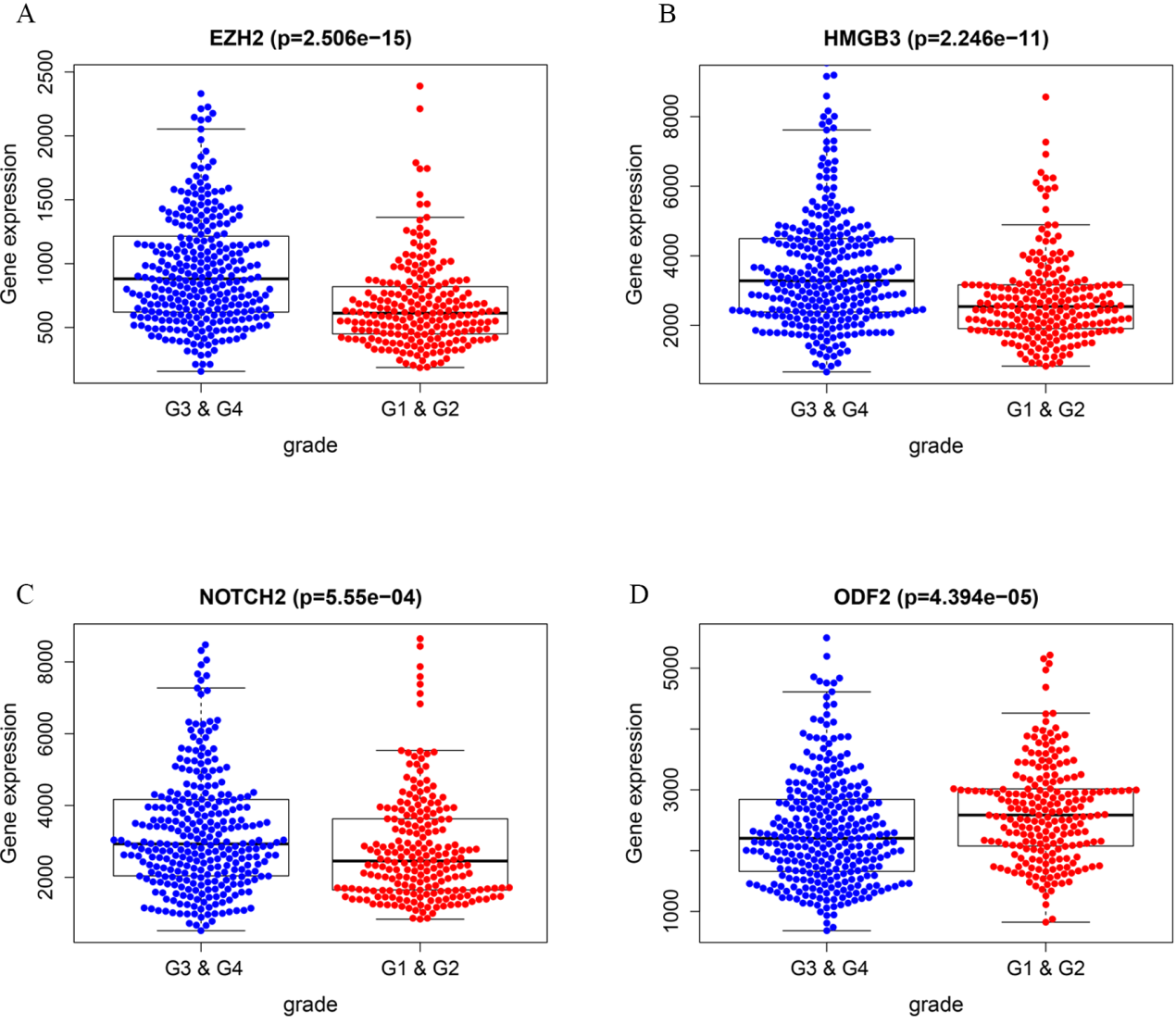
**

**Figure S2** Expression levels of EZH2, HMGB3, NOTCH2 and ODF2 in different grade group. (A) EZH2, (B) HMGB3, (C) NOTCH2, (D) ODF2.
