# Supplementary material for: Establishment of a novel cell cycle-related prognostic signature predicting prognosis in patients with endometrial cancer
Source: Cancer Cell Int. 2020 Jul 20;20:329. doi: 10.1186/s12935-020-01428-z (PMC7372883; doi:10.1186/s12935-020-01428-z)
Supplement: Supplementary file 4 — Additional file 4: Figure S3. (A-B) Expression levels of NOTCH2 and ODF2 in different histological type, (C) expression level of ODF2 in different age group. [file 12935_2020_1428_MOESM4_ESM.docx]

**
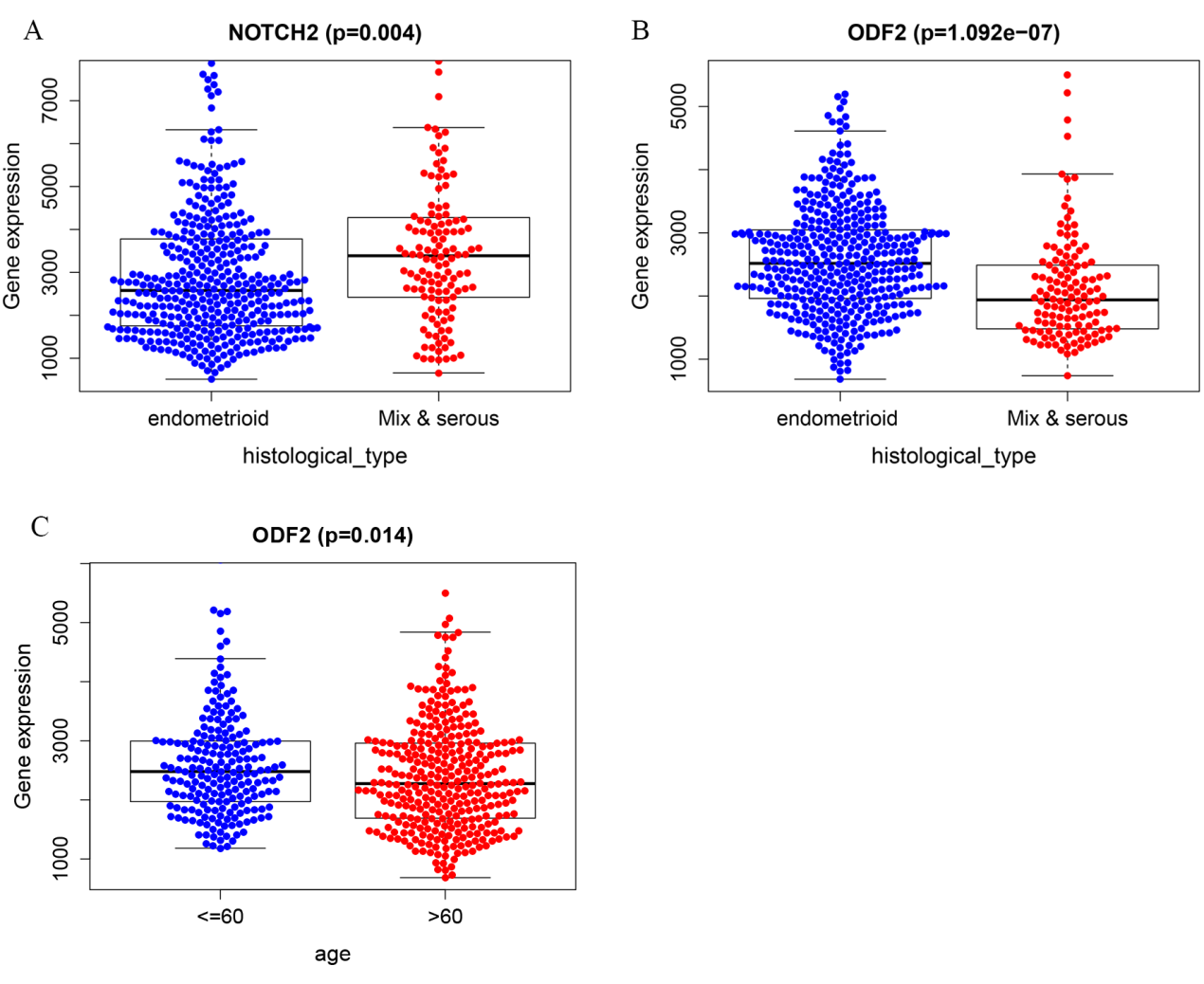
Figure S3** (A-B) Expression levels of NOTCH2 and ODF2 in different histological type, (C) expression level of ODF2 in different age group.
