# Supplementary material for: Establishment of a novel cell cycle-related prognostic signature predicting prognosis in patients with endometrial cancer
Source: Cancer Cell Int. 2020 Jul 20;20:329. doi: 10.1186/s12935-020-01428-z (PMC7372883; doi:10.1186/s12935-020-01428-z)
Supplement: Supplementary file 5 — Additional file 5: Figure S5. Survival time of patients in high-risk and low-risk group of different subgroups. (A) endometrioid subgroup, (B) grade G1&G2 subgroup, (C) grade G3&G4 subgroup, (D) stage III & stage IV subgroup, (E) tumor free subgroup, (F) age>60 subgroup, (G) age≤60 subgroup. [file 12935_2020_1428_MOESM5_ESM.docx]

**
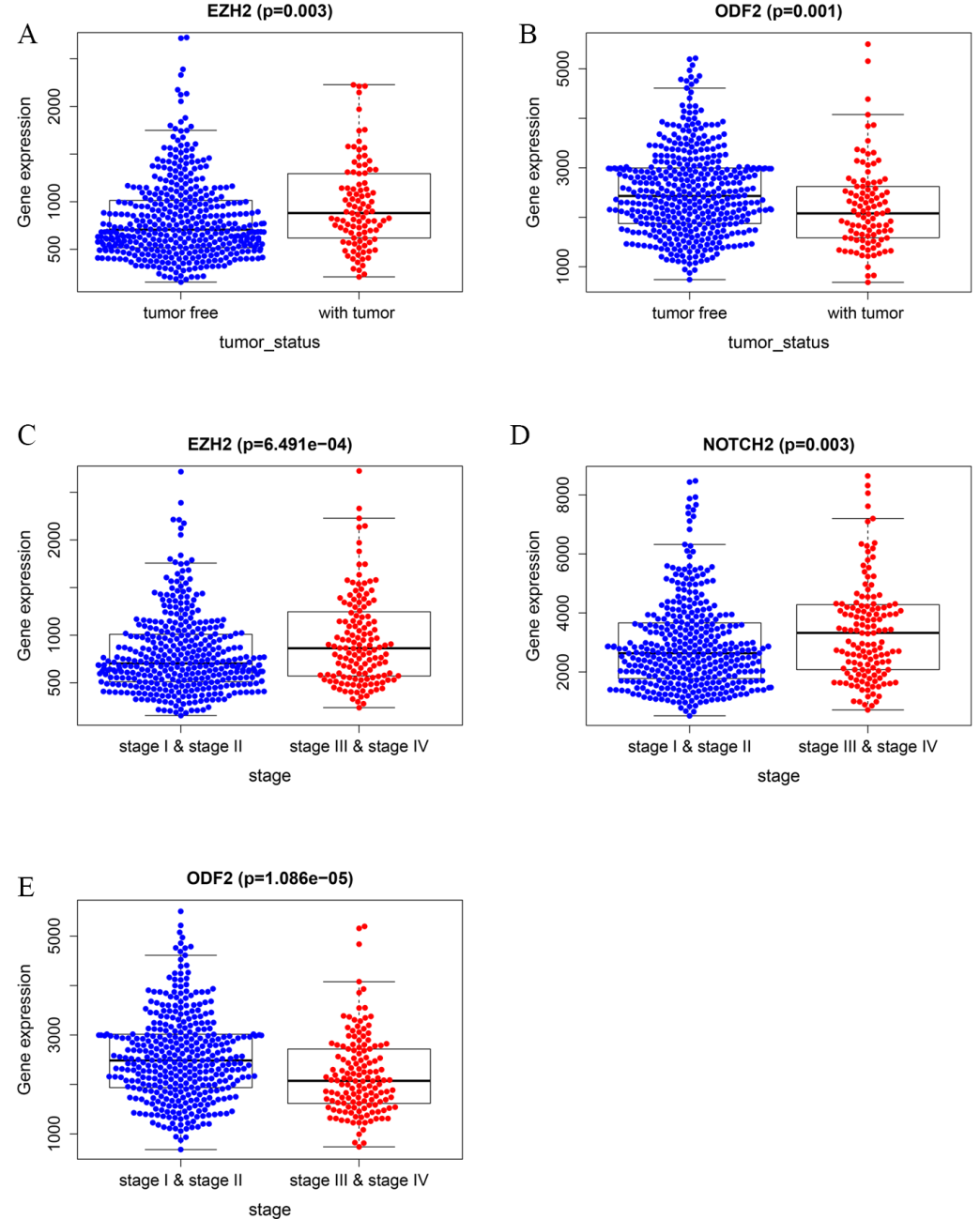
**

**Figure S4** (A-B) Expression levels of EZH2 and ODF2 in different tumor status group, (C-E) expression levels of EZH2, NOTCH2 and ODF2 in different stage group.
