# Supplementary material for: Establishment of a novel cell cycle-related prognostic signature predicting prognosis in patients with endometrial cancer
Source: Cancer Cell Int. 2020 Jul 20;20:329. doi: 10.1186/s12935-020-01428-z (PMC7372883; doi:10.1186/s12935-020-01428-z)
Supplement: Supplementary file 7 — Additional file 7: Figure S6. Principal component analysis of the training cohort, the testing cohort, and the entire EnCa cohort. (A) The training cohort, (B) The testing cohort, (C) The entire cohort. [file 12935_2020_1428_MOESM7_ESM.docx]

**
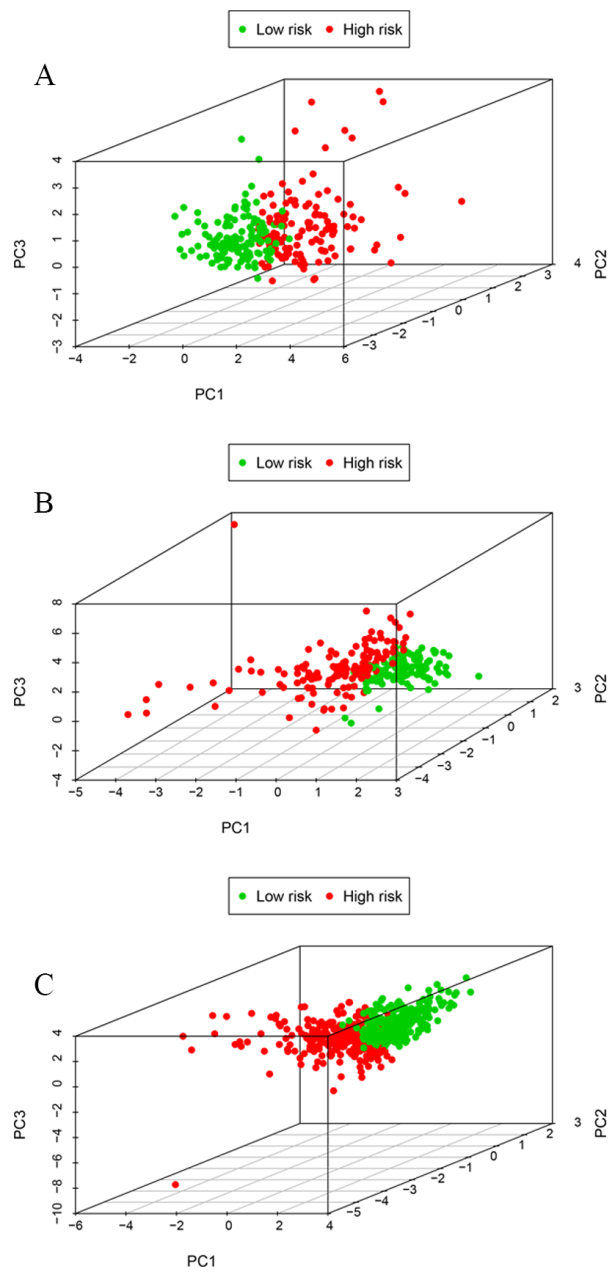
**

**Figure S6** Principal component analysis of the training cohort, the testing cohort, and the entire EnCa cohort. (A) The training cohort, (B) The testing cohort, (C) The entire cohort.
