# Supplementary material for: Establishment of a novel cell cycle-related prognostic signature predicting prognosis in patients with endometrial cancer
Source: Cancer Cell Int. 2020 Jul 20;20:329. doi: 10.1186/s12935-020-01428-z (PMC7372883; doi:10.1186/s12935-020-01428-z)
Supplement: Supplementary file 8 — Additional file 8: Figure S7. The gene mutation overview of 5 prognostic cell cycle-related genes in the TCGA EnCa patients. (A) Five genes were altered in 89 (16%) of the 547 patients/548 samples. (B) The summary of mutation types of 5 genes in EnCa patients. [file 12935_2020_1428_MOESM8_ESM.docx]

**
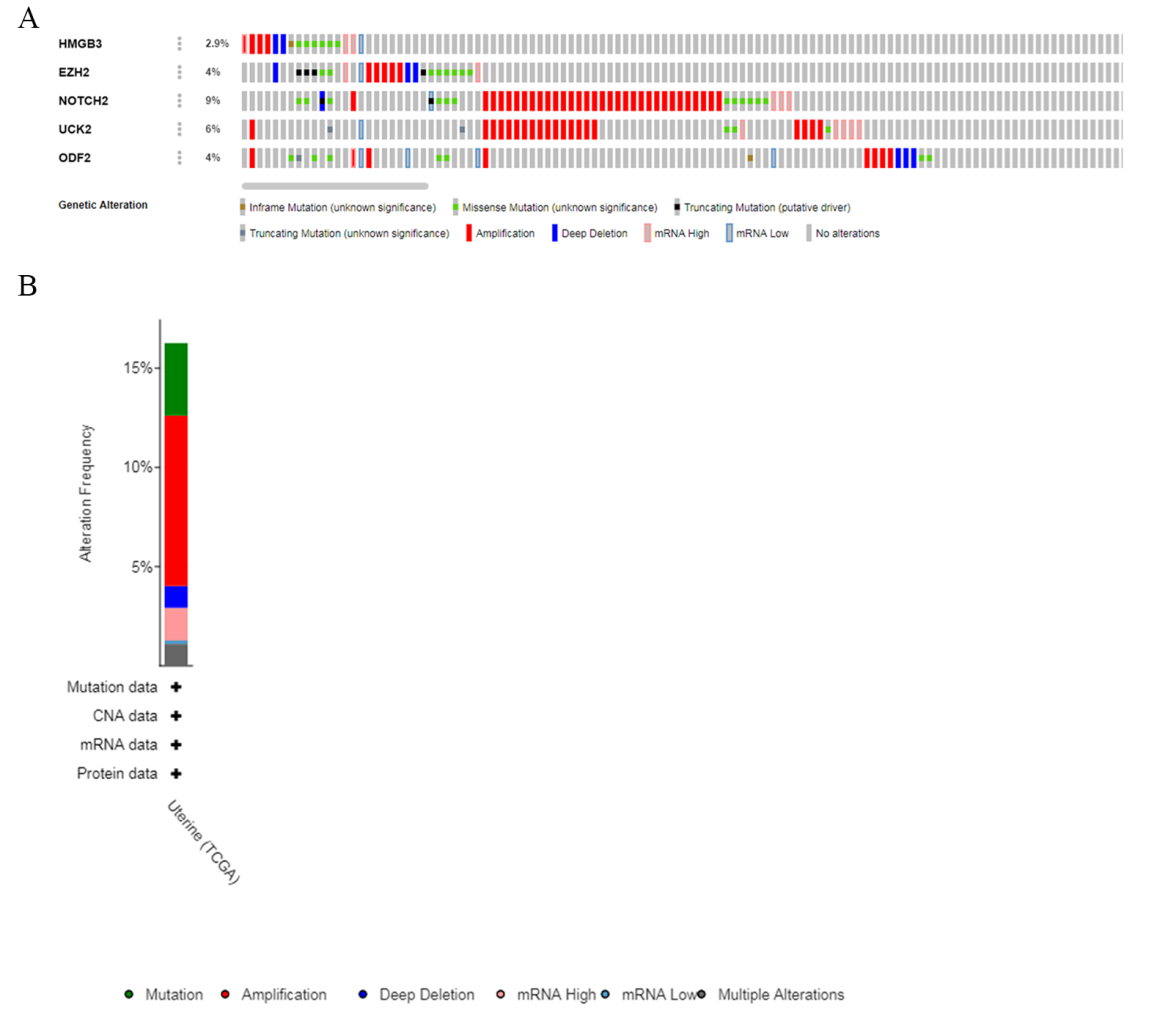
**

**Figure S7** The gene mutation overview of 5 prognostic cell cycle-related genes in the TCGA EnCa patients. (A) Five genes were altered in 89 (16%) of the 547 patients/548 samples. (B) The summary of mutation types of 5 genes in EnCa patients.
