# Supplementary material for: Establishment of a novel cell cycle-related prognostic signature predicting prognosis in patients with endometrial cancer
Source: Cancer Cell Int. 2020 Jul 20;20:329. doi: 10.1186/s12935-020-01428-z (PMC7372883; doi:10.1186/s12935-020-01428-z)
Supplement: Supplementary file 9 — Additional file 9: Figure S8. AUC value was used to identify the diagnostic efficacy of distinguishing normal and cancerous tissues. [file 12935_2020_1428_MOESM9_ESM.docx]

**
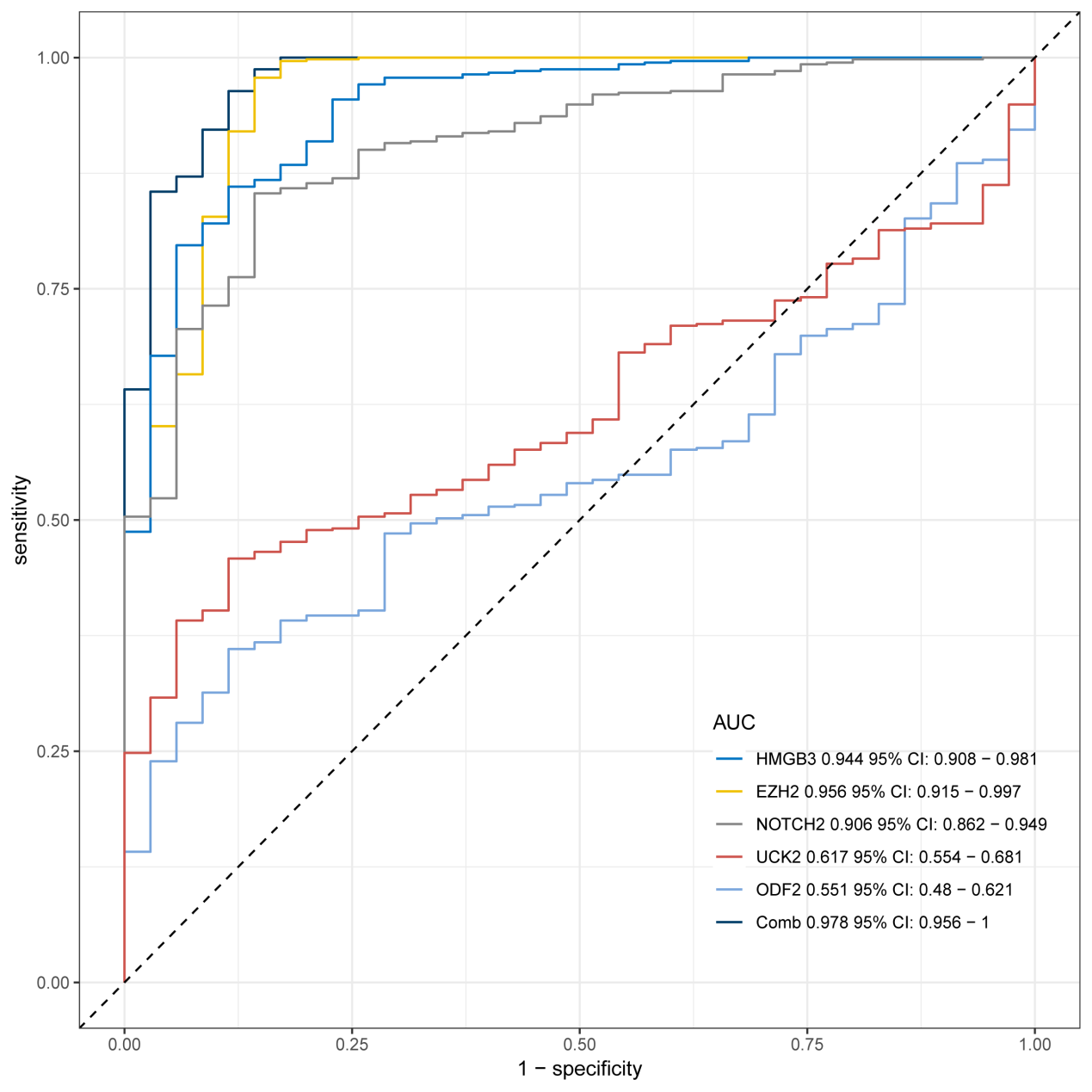
**

**Figure S8** AUC value was used to identify the diagnostic efficacy of distinguishing normal and cancerous tissues.
