# Supplementary material for: Establishment of a novel cell cycle-related prognostic signature predicting prognosis in patients with endometrial cancer
Source: Cancer Cell Int. 2020 Jul 20;20:329. doi: 10.1186/s12935-020-01428-z (PMC7372883; doi:10.1186/s12935-020-01428-z)
Supplement: Supplementary file 10 — Additional file 10: Figure S9. Kaplan-Meier curves of the EZH2, HMGB3, NOTCH2 and ODF2. The yellow line indicates samples with highly expressed genes (above best-separation value), and the green line designates the samples with lowly expressed genes (below best-separation value) [file 12935_2020_1428_MOESM10_ESM.docx]

**
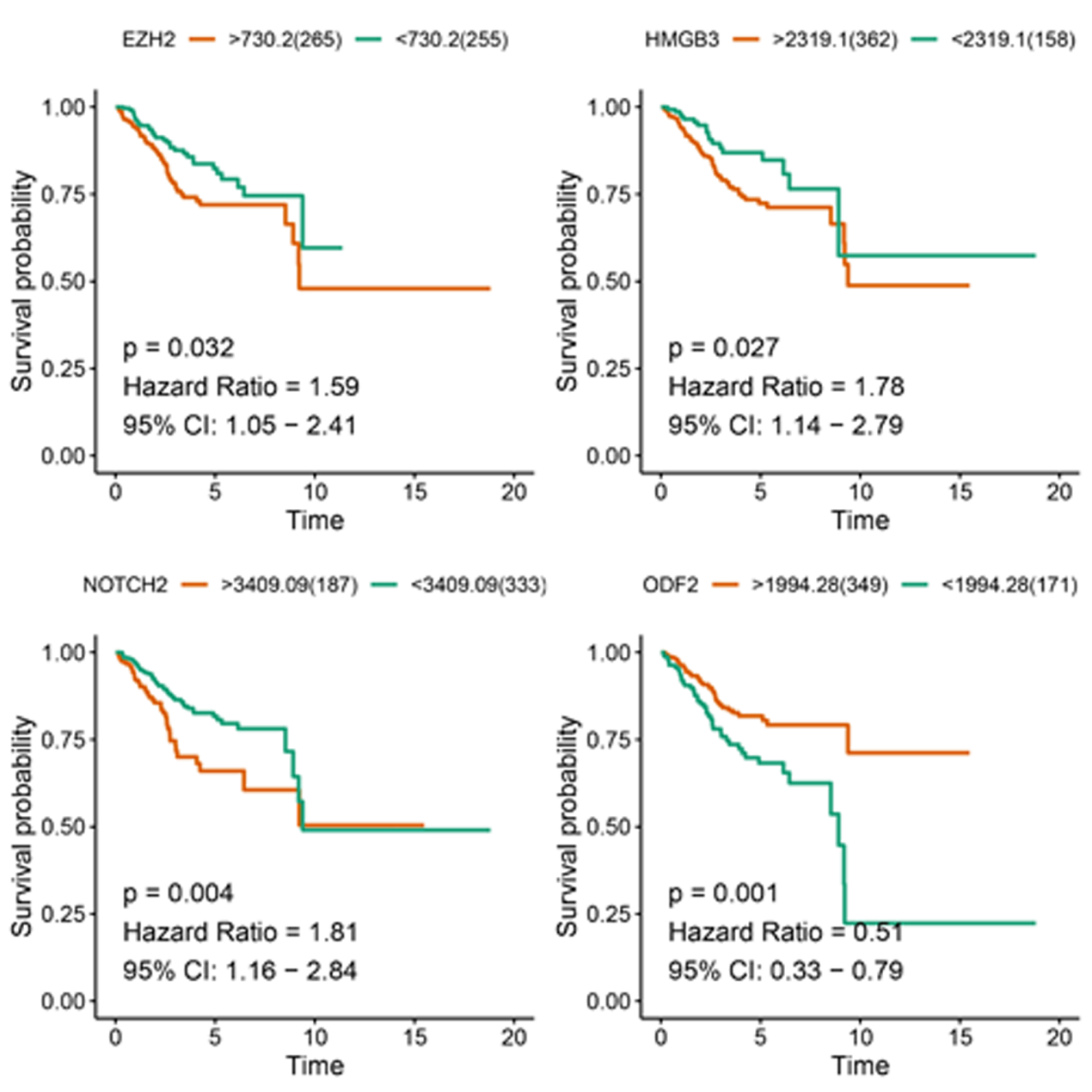
**

**Figure S9** Kaplan-Meier curves of the EZH2, HMGB3, NOTCH2 and ODF2. The yellow line indicates samples with highly expressed genes (above best-separation value), and the green line designates the samples with lowly expressed genes (below best-separation value)
