# Supplementary material for: Establishment of a novel cell cycle-related prognostic signature predicting prognosis in patients with endometrial cancer
Source: Cancer Cell Int. 2020 Jul 20;20:329. doi: 10.1186/s12935-020-01428-z (PMC7372883; doi:10.1186/s12935-020-01428-z)
Supplement: Supplementary file 11 — Additional file 11: Figure S10 Alteration landscape for EnCa samples with high risk score and low-risk in the TCGA cohort. Lower rates of PTEN mutation, TTN mutation, ARID1A mutation, PIK3R1 mutation, ZFHX3 mutation and PIK3CA mutation in EnCa with high-risk score were found compared with EnCa with low risk score. [file 12935_2020_1428_MOESM11_ESM.docx]

**
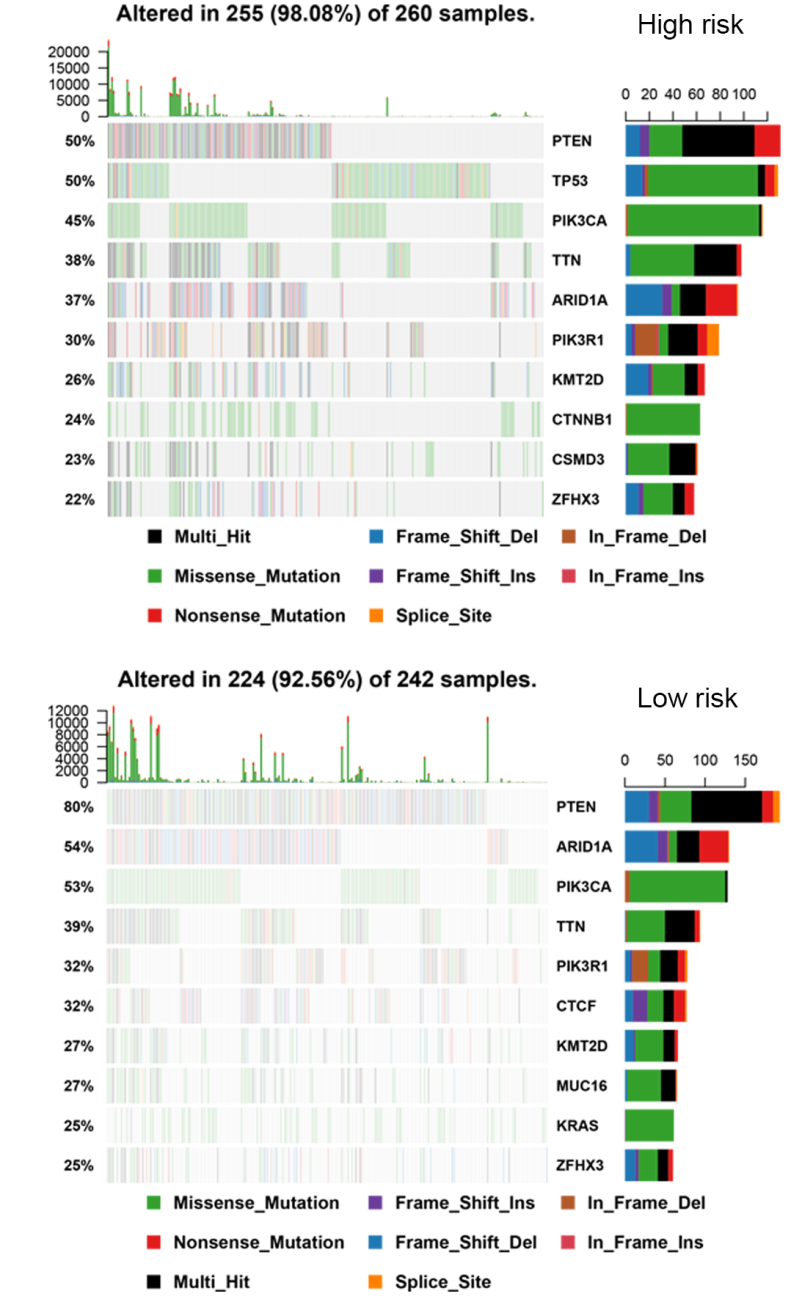
**

**Figure S10** Alteration landscape for EnCa samples with high risk score and low-risk in the TCGA cohort. Lower rates of PTEN mutation, TTN mutation, ARID1A mutation, PIK3R1 mutation, ZFHX3 mutation and PIK3CA mutation in EnCa with high-risk score were found compared with EnCa with low risk score.
